# Astragalus polysaccharides attenuate PCV2 infection by inhibiting endoplasmic reticulum stress *in vivo* and *in vitro*

**DOI:** 10.1038/srep40440

**Published:** 2017-01-10

**Authors:** Hongxia Xue, Fang Gan, Gang Qian, Junfa Hu, Shu Hao, Jing Xu, Xingxiang Chen, Kehe Huang

**Affiliations:** 1 College of Veterinary Medicine, Nanjing Agricultural University, Nanjing 210095, Jiangsu Province, China

## Abstract

This study explored the effects of Astragalus polysaccharide (APS) on porcine circovirus type 2 (PCV2) infections and its mechanism *in vivo* and vitro. First, fifty 2-week-old mice were randomly divided into five groups: a group without PCV2 infection and groups with PCV2 infections at 0, 100, 200 or 400 mg/kg APS treatments. The trial lasted for 28 days. The results showed that APS treatments at 200 and 400 mg/kg reduced the pathological injury of tissues, inhibited PCV2 infection and decreased glucose-regulated protein 78 (GRP78) and GADD153/CHOP gene mRNA and protein expression significantly (P < 0.05). Second, a study on endoplasmic reticulum stress mechanism was carried out in PK15 cells. APS treatments at 15 and 45 μg/mL significantly reduced PCV2 infection and GRP78 mRNA and protein expression (P < 0.05). Tunicamycin supplementation increased GRP78 mRNA and protein expression and significantly attenuated the APS-induced inhibition of PCV2 infection (P < 0.05). Tauroursodeoxycholic acid supplementation decreased GRP78 mRNA and protein expression and significantly inhibited PCV2 infection (P < 0.05). In addition, fifty 2-week-old mice were randomly divided into five groups: Con, PCV2, APS + PCV2, TM + PCV2 and TM + APS + PCV2. The results were similar to those in PK15 cells. Taken together, it could be concluded that APS suppresses PCV2 infection by inhibiting endoplasmic reticulum stress.

Porcine circovirus type 2 (PCV2) is one of the smallest DNA viruses infecting mammals^1^ and belongs to the family Circoviridae and genus Circovirus. PCV2 is currently regarded as the essential factor responsible for causing porcine circovirus-associated diseases (PCVADs) in pigs^2^, which include postweaning multisystemic wasting syndrome (PMWS)^3^, porcine respiratory disease complex (PRDC) and porcine dermatitis and nephropathy syndrome (PDNS) and reproduction disorders 4. PCVAD has caused huge economic losses for the global swine industry^5^,^6^,^7^.

Since late 2009, commercial vaccines against this virus have been used in pigs^8^,^9^. However, PCV2 infection status can be quite complex clinically at present, and the widespread use of vaccination on farms will probably shift general pig health from a period of severe worldwide clinical outbreaks to self-limiting subclinical infections with occasional outbreaks^10^,^11^,^12^. A PCV2 vaccine “might not work as expected” or “may fail”. Trying to reduce our dependence on vaccines, we should identify other effective medicines to reduce the growth retardation and infection caused by PCV2.

Astragalus polysaccharide (APS) is one of the main constituents of Astragalus, a traditional Chinese medicine that has been studied in depth and is widely used in clinical settings^13^,^14^,^15^. APS has been proved to have various biological activities, including antioxidant, immuno-modulatory, anti-tumour, anti-inflammatory, and antiviral activities^16^,^17^,^18^,^19^,^20^,^21^ among others. Biological polysaccharides have been identified to have effects on the oxidative stress induced by PCV2 *in vivo* and *in vitro*^22^,^23^. A previous study showed that APS has a protective effect against PCV2 replication *in vitro*^24^. While APS is also used in actual pig production, the mechanisms underlying the above effects are unclear *in vivo*.

We have demonstrated that APS inhibits PCV2 replication through antioxidant activity and inhibition of the NF-κ B pathway *in vitro*^24^. Studies have indicated that APS has promising applications in the treatment of type 2 diabetes by enhancing the ability of cells to adapt to endoplasmic reticulum (ER) stress^25^,^26^. Previous studies have shown that oxidative stress enhances PCV2 replication^27^,^28^. PCV2 replication increases oxidative stress^29^,^30^, and oxidative stress induces ER stress^31^. Viruses rely on host chaperones to support their infection. In particular, the ER resident chaperones play key roles in synthesizing and processing viral proteins. The influx of a large amount of foreign proteins exhausts the folding capacity in the ER and triggers the unfolded protein response (UPR). Studies have determined that UPR elements could be important therapeutic targets for decreasing DENV multiplication^32^. A previous study showed that ER stress signalling is important to viral replication by addressing the earliest phase of autophagy induced by ER stress to limit virus production and the survival of dengue-infected cells^33^. Few studies have demonstrated that APS can inhibit PCV2 infection through reducing ER stress.

Therefore, the objectives of the current study were to explore the ability of APS to inhibit PCV2 infection and its relationship to ER stress mechanisms using PCV2 infection models in mice and PK15 cells. We hypothesized that APS inhibits PCV2 infection by suppressing ER stress.

## Results

### The effects of different APS concentrations on the abatement of pathological changes induced by PCV2 infection

**Figure 1:**
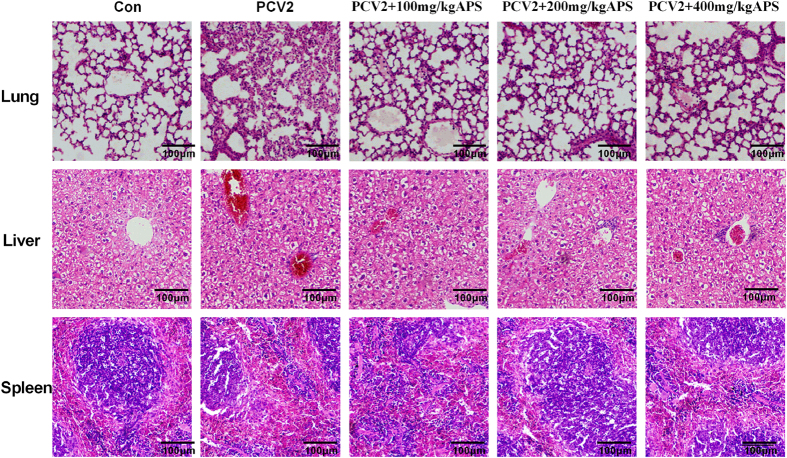
The effects of different concentrations of APS on tissue histopathology in mice infected with PCV2. HE stain.

The changes in pathological tissue sections stained with H&E are shown in Fig. 1. Compared with the blank control group, the tissue surrounding the lung alveoli and bronchioles showed stromal hyperplasia and pulmonary haemorrhage, and there were a large number of peripheral alveolar infiltrates, etc., while APS supplementation reduced pathological damage to the lung. Hepatic necrosis and congestion were serious, while the APS treatment groups showed reduced pathological damage to the liver. The characteristics of the red pulp in the spleen did not change, and the effect of APS was not obvious.

### APS inhibits PCV2 infection *in vivo*

**Figure 2:**
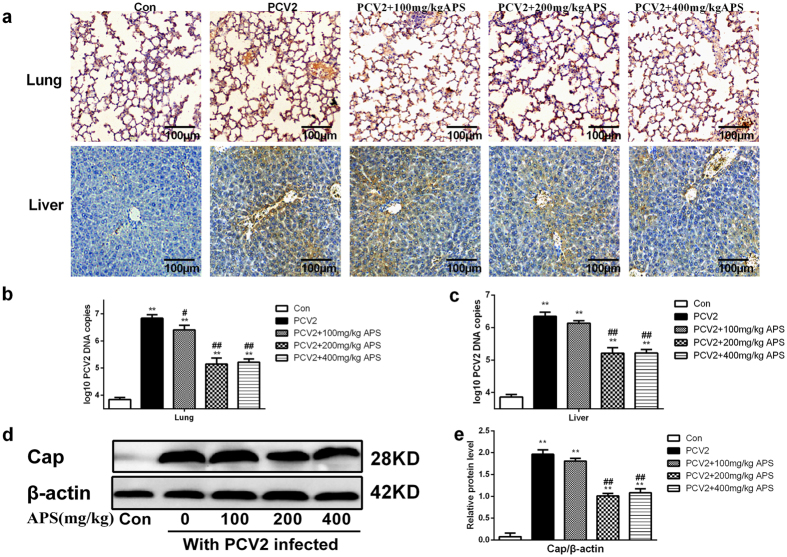
APS inhibits PCV2 infection *in vivo*. The effects of different concentrations APS on the IHC results in mice infected with PCV2 (**a**). Effects of APS on PCV2 DNA copies in the lung (**b**) and liver (**c**). Effect of APS on Cap protein expression in the lung, as determined by western blot (**d**,**e**). Western blots were analysed under the same experimental conditions. Data are presented as the means ± SD. For all groups, significance was calculated relative to the control, *p < 0.05 and **p < 0.01. For the groups with PCV2 infection, significance was calculated relative to the PCV2 control, ^#^p < 0.05 and ^##^p < 0.01.

As shown in Fig. 2a, the brown areas of immunohistochemical staining indicated virus infection via microscopic observation. Compared with the control group, the lungs and livers of the mice infected with PCV2 had PCV2 infection. Compared with the PCV2 control group, different concentrations of APS weakened PCV2 infection to varying degrees.

The number of PCV2 DNA copies in the lungs and livers of mice were assayed with real-time PCR. As shown in Fig. 2b and c, the number of PCV2 DNA copies was significantly higher in the lungs and livers of PCV2-infected mice (P < 0.05) than the control group. Furthermore, different concentrations of APS significantly reduced the number of PCV2 DNA copies (P < 0.05).

The results showed that the pathological damage to the lung tissues of PCV2-infected mice was more serious, and the inhibitory effects of APS on PCV2 infection were more obvious in lung tissues. Therefore, we used the lung tissue to determine Cap protein expression levels, which would verify the inhibitory effects of APS on PCV2 infection. As shown in Fig. 2d and e, APS can actually reduce Cap protein expression. Therefore, these results indicated that APS inhibited PCV2 infection in mice.

### APS reduces oxidative stress in PCV2-infected mice

To verify that PCV2 infection can increase the oxidative stress markers in the serum of mice and that APS can decrease the generation of oxidative stress, mice were treated as described in the Methods section, blood was collected and serum was separation. The lung and liver tissues were treated as described in the Methods. The changes in oxidative stress markers in the serum and tissues of mice were detected according to the kit instructions.

**Figure 3:**
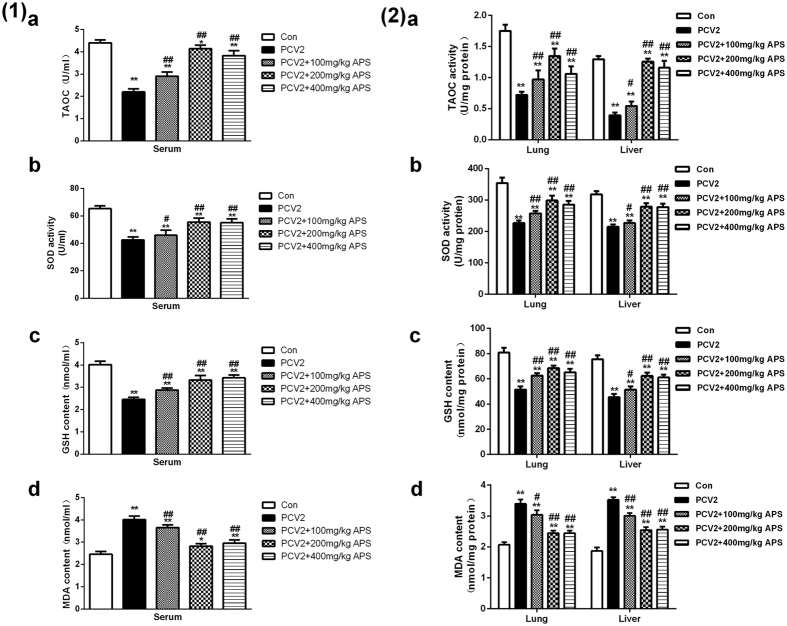
APS decreases oxidative stress in the serum and tissues of mice infected with PCV2. TAOC activity (**a**), SOD activity (**b**), GSH content (**c**) and MDA content (**d**) in the serum (1) and the tissues (2) were determined following the instructions of the kits. The values shown are the means ± SD from three independent experiments. Groups were compared with a one-way ANOVA followed by the least-significant difference test. For all groups, significance was calculated relative to the cell control, *p < 0.05 and **p < 0.01. For PCV2 infection, significance was calculated relative to the virus control, ^#^p < 0.05 and ^##^p < 0.01.

The results are shown in Fig. 3, PCV2 infection significantly decreased T-AOC activity, SOD activity and GSH content compared with the control groups, and APS at 100, 200, 400 mg/kg reversed these changes (Fig. 3(1) and (2)a,b and c). PCV2 infection significantly increased the MDA content compared with the control group (P < 0.05), and APS at 100, 200, 400 mg/kg reversed the increase in MDA content (Fig. 3(1) and (2)d). APS at 200 mg/kg had the largest effect on these parameters. These results suggest that APS could alleviate the oxidative stress in PCV2-infected mice.

### APS attenuates endoplasmic reticulum stress in PCV2-infected mice

**Figure 4:**
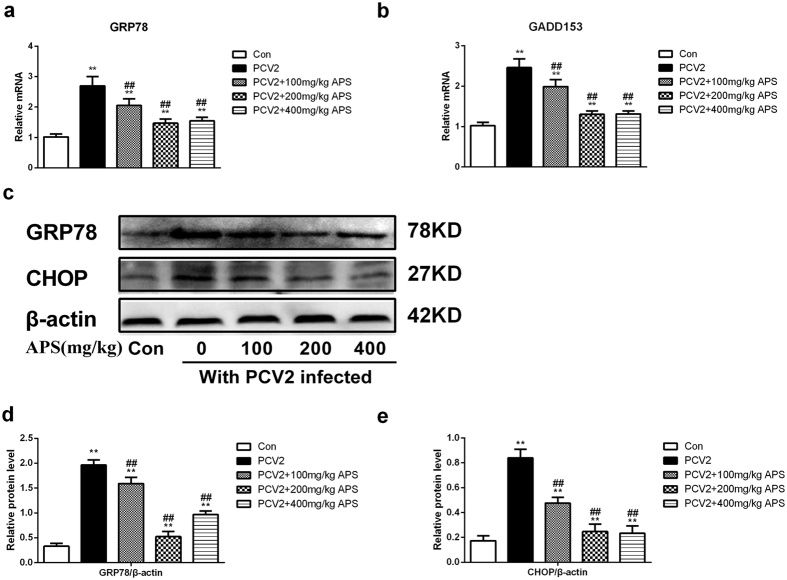
APS attenuates endoplasmic reticulum stress induced by PCV2 infection in mice. Lung tissues were assayed for the GRP78 mRNA level (**a**) and CHOP mRNA level (**b**); actin, GRP78 and CHOP protein expression were determined by western blot (**c**,**d** and **e**) as described in the Materials and Methods section. Western blots were analysed under the same experimental conditions. The values shown are the means ± SD from three independent experiments. The groups were compared with a one-way ANOVA followed by the least-significant difference test. For all groups, significance was calculated relative to the control, *p < 0.05 and **p < 0.01. For the PCV2 groups, significance was calculated relative to the cells treated with PCV2, ^#^p < 0.05 and ^##^p < 0.01.

We detected large amount of PCV2 in the tissues through RT-PCR, western blotting (WB) and IHC; here, we attempt to explain part of the mechanism of ER stress in the lung. First, we detected the mRNA expression levels of genes associated with endoplasmic reticulum stress (GRP78 and GADD153/CHOP) in the lung. As shown in Fig. 4a and b, PCV2 infection increased the relative mRNA levels of GRP78 and CHOP, and APS at 100, 200, 400 mg/kg blocked the increases induced by PCV2 infection. In addition, we examined the expression of these proteins using WB; the results are shown in Fig. 4c,d and e. PCV2 infection increased the protein expression of GRP78 and CHOP, and different concentrations of APS could reverse these changes. The effects of APS on GRP78 expression were larger than the effects on CHOP expression. These results suggest that APS reduces the ER stress induced by PCV2 infection in the lungs of mice.

### APS attenuates PCV2 replication and endoplasmic reticulum stress in PCV2-infected PK15 cells

We established five groups, namely Con (nothing added, the control group), PCV2 (positive control group), PCV2 + 5 μg/mL APS group, PCV2 + 15 μg/mL APS group and PCV2 + 45 μg/mL APS group, to verify whether PCV2 infection can cause endoplasmic reticulum stress and determine whether the effects can be weakened by APS in PK15 cells. The methods were as follows: cells were seeded in 12-well plates and 96-well plates and were incubated with APS at 0, 5, 15, 45 μg/mL for 12 h, then cells were or were not infected with 1 MOI PCV2 and cultured for an additional 48 h.

**Figure 5:**
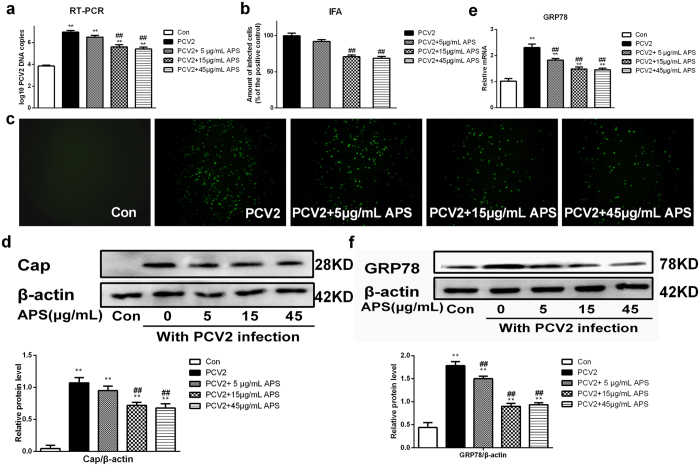
APS attenuates endoplasmic reticulum stress induced by PCV2 infection in PK15 cells. The number of viral DNA copies (**a**), the relative proportion of PCV2-infected cells (**b** and **c**), the relative expression of the Cap protein (western blot) (**d**), and the GRP78 mRNA level (**e**) and relative protein expression (western blot) (**f**) were assayed as described in the Materials and Methods section. Western blots were analysed under the same experimental conditions. The values shown are the means ± SD from three independent experiments. The groups were compared by a one-way ANOVA followed by the least-significant difference test. For all groups, significance was calculated relative to the control, *p < 0.05 and **p < 0.01. For the PCV2 groups, significance was calculated relative to the PCV2 treated cells, ^#^p < 0.05 and ^##^p < 0.01.

Cells were collected to determine the number of PCV2 DNA copies (cells in 12-well plates). As shown in Fig. 5a, different concentrations of APS significantly reduced the number of PCV2 DNA copies (P < 0.05). IFA (cells in 96-well plates) was used to detect the number of PCV2 positive infected cells. As shown in Fig. 5b and c, different concentrations of APS reduced the number of PCV2 positive infected cells substantially. Meanwhile, western blot was used to detect Cap protein expression; as shown in Fig. 5d, different concentrations of APS significantly reduced Cap protein expression (P < 0.05). The results of the effects of APS on ER stress in PCV2-infected cells are shown in Fig. 5e and f; APS reduced GRP78 mRNA and protein expression significantly (P < 0.05) *in vitro*.

### TM attenuates the effect of APS on PCV2 infection *in vitro*

To verify whether APS inhibited PCV2 infection by endoplasmic reticulum stress, we established five groups, including Con (nothing added, the control group), PCV2 (positive control group), PCV2 + APS group (APS, 15 μg/mL), PCV2 + TM group (TM, 1.0 μg/mL TM, ER stress activator) and PCV2 + APS + TM group (APS, 15 μg/mL; TM, 1.0 μg/mL TM), and used TM an endoplasmic reticulum stress activator.

**Figure 6:**
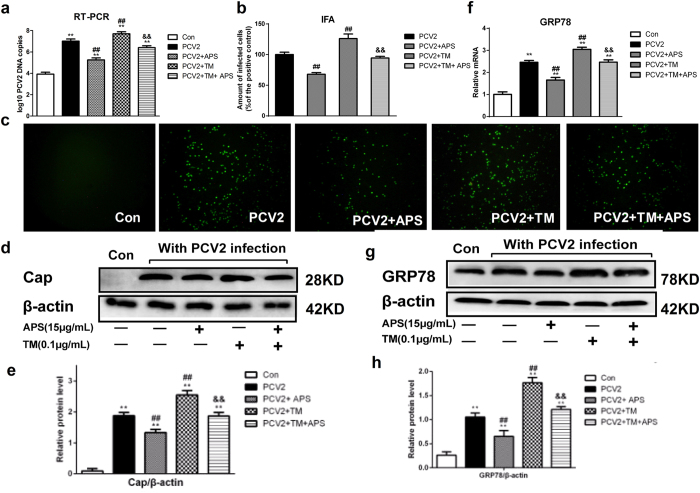
TM attenuates the effect of APS on PCV2 replication *in vitro*. Cell samples were assayed for the number of PCV2 DNA copies (**a**), the relative proportion of PCV2-infected cells (**b** and **c**), the relative expression of the Cap protein (western blot) (**d** and **e**), GRP78 mRNA level (**f**) and protein expression (western blot) (**g** and **h**), as described in the Materials and Methods section. Western blots were analysed under the same experimental conditions. The values shown are the means ± SD from three independent experiments. Groups were compared with a one-way ANOVA followed by the least-significant difference test. For all groups, significance was calculated relative to the control, *p < 0.05 and **p < 0.01. For the PCV2 groups, significance was calculated relative to the cells treated with only PCV2, ^#^p < 0.05 and ^##^p < 0.01. For the APS groups, significance was calculated relative to the untreated cells with TM, ^&^p < 0.05 and ^&&^p < 0.01.

The results are shown in Fig. 6. TM increased PCV2 replication and GRP78 mRNA and protein expression. TM reversed the decreasing effects of APS on PCV2 DNA copy number (Fig. 6a), the number of PCV2-infected positive cells (Fig. 6b and c) and Cap protein expression (Fig. 6d). These results suggest that TM attenuates the inhibiting effects of APS on PCV2 replication *in vitro*.

### Tauroursodeoxycholic acid (TUDCA) attenuates PCV2 replication and endoplasmic reticulum stress in PCV2-infected PK15 cells

We established four groups, namely Con (nothing added, the control group), a 0.3 mg/mL TUDCA group (ER stress inhibitor), a PCV2 group, and a PCV2 + 0.3 mg/mL TUDCA group, to verify whether TUDCA could decrease endoplasmic reticulum stress and inhibit PCV2 replication in PK15 cells. The methods were as follows: cells were seeded in 12-well plates and 96-well plates and incubated with 0.3 mg/mL TUDCA for 12 h, then cells were or were not infected with 1 MOI PCV2 and cultured for an additional 48 h.

**Figure 7:**
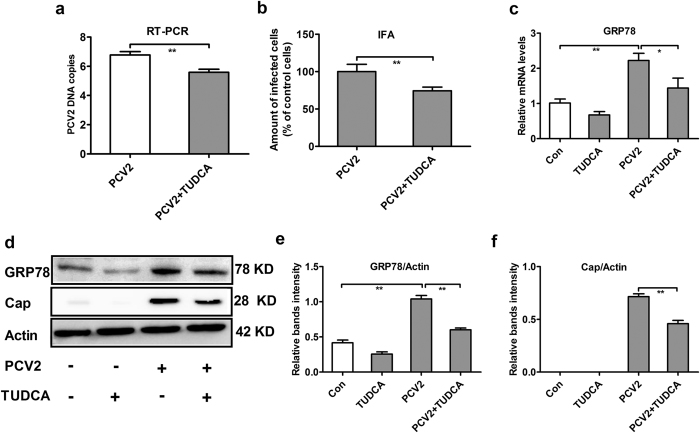
TUDCA attenuates PCV2 infection and endoplasmic reticulum stress in PK15 cells. The number of viral DNA copies (**a**), the relative proportion of PCV2-infected cells and PCV2-infected cells (**b**), the relative expression of the Cap protein (western blot) (**d**,**f**), the GRP78 mRNA level (**c**) and the relative protein expression (western blot) (**d**,**e**) were assayed as described in the Materials and Methods section. Western blots were analysed under the same experimental conditions. The values shown are the means ± SD from three independent experiments. Groups were compared with a t-test or a one-way ANOVA followed by a least-significant difference test. *p < 0.05 indicates significance and **p < 0.01 indicates extreme significance.

Cells were collected to detect the number of PCV2 DNA copies (cells in 12-well plates). As shown in Fig. 7a, TUDCA significantly reduced the number of PCV2 DNA copies (P < 0.05). IFA (cells in 96-well plates) was used to detect the number of PCV2 positive infected cells. As shown in Fig. 7b, TUDCA significantly reduced the number of PCV2 positive infected cells; meanwhile, western blot was used to detect Cap protein expression. As shown in Fig. 7d–f, TUDCA significantly reduced Cap protein expression (P < 0.05). The results of the effects of TUDCA on ER stress in PCV2-infected cells are shown in Fig. 7c,d and f; TUDCA reduced GRP78 mRNA and protein expression significantly (P < 0.05).

### TM attenuates the effects of APS on PCV2 infection *in vivo*

To further verify whether the APS mediated suppression of PCV2 infection in mice occurs through inhibiting endoplasmic reticulum stress, we used TM, an activator of ERS, to inject into mice, and the mouse feeding and sample collection were performed as described in the second mouse trial section of the Materials and Methods.

**Figure 8:**
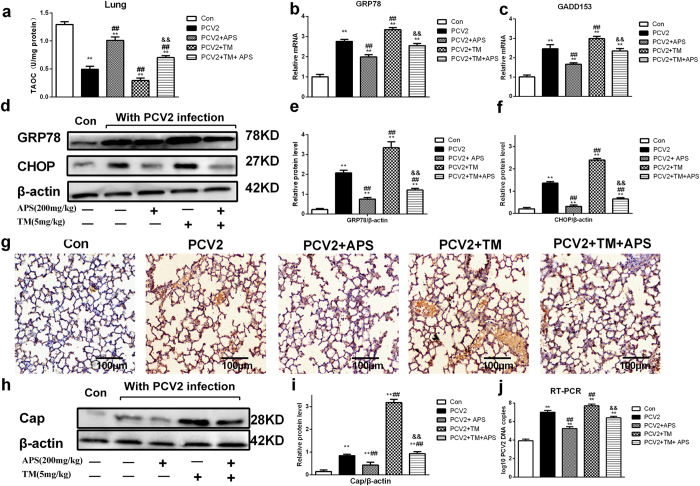
TM attenuates the effect of APS on PCV2 infection in mice. Lung tissues were assayed for TAOC activity (**a**); GRP78 gene expression (**b**); CHOP mRNA level (**c**); and relative GRP78 and CHOP protein expression with western blotting (**d**,**e** and **f**). The sections showing PCV2 Cap protein appeared brown after immunohistochemical staining (**g**). The relative Cap protein expression (western blotting) (**h** and **i**) and PCV2 DNA copies (**j**) were determined as described in the Materials and Methods section. Western blots were analysed under the same experimental conditions. The values shown are the means ± SD from three independent experiments. The groups were compared with a one-way ANOVA followed by a least-significant difference test. For all groups, significance was calculated relative to the control, *p < 0.05 and **p < 0.01. For the PCV2 groups, significance was calculated relative to the cells treated with only PCV2, ^#^p < 0.05 and ^##^p < 0.01. For the APS groups, significance was calculated relative to the untreated cells with TM, ^&^p < 0.05 and ^&&^p < 0.01.

The results are shown in Fig. 8. TM treatment decreased T-AOC activity (Fig. 8a), increased GRP78 and CHOP mRNA and protein expression (Fig. 8b–f), and increased PCV2 infection, as demonstrated by increases in Cap protein expression (Fig. 8g–i) and the number of PCV2 DNA copies (Fig. 8j). APS increased T-AOC activity, decreased GRP78 and CHOP mRNA and protein expression significantly (P < 0.05), and decreased PCV2 infection. Furthermore, TM reversed the above changes induced by APS. These results suggest that the inhibitory effect of APS on PCV2 replication *in vitro* is mediated through ERS.

## Discussion

PCV2 infection is known as the main factor in inducing PCVAD, which causes huge economic losses in the swine industry^6^. Our previous study showed that APS could inhibit PCV2 replication *in vitro*^24^. We researched the effects of APS on PCV2 infection by observing the animal weight and pathological changes and detecting the number of PCV2 DNA copies and Cap protein expression (IHC and western blot) in mice. As shown in Figs 1 and 2a, obvious changes in the lung and liver tissues of PCV2-infected mice were observed; these results are similar to reports in which a mouse model was used to study PCV2 infection by detecting viral distribution and lesions^34^. As shown in Fig. 2b and c, the number of PCV2 DNA copies was greater in PCV2-infected mice, and the effects of APS were more obvious in the lung. Therefore, we selected the lung tissue to determine Cap protein expression levels, verifying the inhibitory effects of APS on PCV2 infection in mice (Fig. 2d and e). Our results showed that APS could inhibit PCV2 infection *in vivo* (Figs 1 and 2), which was consistent with a previous *in vitro* study^24^.

Some reports of the anti-virus effects of the polysaccharide researched the mechanism underlying the strengthening of the immune system^35^,^36^. However, our previous study showed that APS suppresses PCV2 infection through its antioxidant activity and inhibiting the NF-κ B pathway *in vitro*^24^. It has been reported that ER stress and/or inflammation may be basic mechanisms that increase the severity or complicate the condition of the disease and oxidative stress^37^. Therefore, we are trying to clarify the mechanism of ER stress from all sides. As shown in Fig. 3, the oxidative stress in mice with PCV2 infection could be changed significantly (P < 0.05), and APS attenuated the oxidative stress induced by PCV2 infection in the serum and tissues.

A fully executed UPR comprises signalling pathways that induce ER stress^37^, increase protein degradation, block new protein synthesis and may eventually activate apoptosis, presenting both opportunities and threats to the virus. Studies have shown that viral replication is associated with ER stress^38^,^39^,^40^. We studied the ER stress situation in lung tissue and the mRNA and protein expression levels of ER stress-related genes. We further analysed the correlation between the expression levels of ER stress-related molecules and PCV2 infections in mice. Moreover, the ER stress-related apoptosis molecule CHOP was activated in PCV2-infected mice. Our results show that PCV2 infection significantly increased (P < 0.01) the expression of the ERS related genes GRP78 and GADD153/CHOP at the mRNA and protein level in mice, which was similar to reports that hepatitis B and C virus-induced hepatitis induced apoptosis, autophagy, and the unfolded protein response^41^. The inhibitory function of APS on PCV2 infection in mice could be mediated by lowering the expression of GRP78 and CHOP. We found that there was a corresponding change in endoplasmic reticulum stress (Fig. 4).

To verify the results in our mouse model, we made the corresponding tests *in vitro*. Our results showed that APS could inhibit PCV2 replication in PK15 cells, consistent with our previous report^24^, as well as an ERS situation (Fig. 5). TM, an activator of ER stress, increased PCV2 replication and reversed the inhibitory effects of APS on PCV2 replication (Fig. 6). TUDCA, an inhibitor of ER stress, decreased GRP78 expression and decreased PCV2 replication (Fig. 7), consistent with other reports that TUDCA significantly inhibited ER stress^42^ and influenza A viral replication^43^. We also sought to determine whether APS inhibited PCV2 infection by attenuating ER stress *in vivo*. To fulfil this objective, we injected TM into the abdominal cavity of mice. Our results showed that TM could induce ER stress, promote PCV2 replication, and reverse the inhibitory effects of APS on PCV2 infection (**8**). The *in vitro* and *in vivo* results were consistent.

This study explored the effects of APS on PCV2 infection by monitoring the endoplasmic reticulum stress chaperone GRP78. However, it is still unclear which specific signalling pathway is at work. Further studies are warranted to elucidate these pathways in relation to the mechanism by which APS inhibits PCV2 infection. In addition, studies have shown that endoplasmic reticulum stress stimulates autophagy, and little of our evaluation of traditional Chinese medicine investigated apoptosis, autophagy, and the UPR, which suggests that more studies on the effects of this polysaccharide with regard to autophagy are needed. Additionally, studies have shown that the medicinal activity of APS can be enhanced through a variety of modifications, such as sulfation, methylation or phosphorylation^44^. This suggests that more studies on the development of the corresponding antiviral drugs are imperative.

In conclusion, the present study verified the inhibitory effects of APS on PCV2 infection and identified part of its mechanism of attenuating ER stress *in vivo* and *in vitro,* contributing to the further investigation of APS in anti-viral research. Our study suggests that APS might be employed to prevent PCV2 infection and as a protective or adjuvant therapeutic drug in the management of PCVAD and might be widely used to aid the effects of anti-viral drugs.

## Materials and Methods

### Preparation of APS, PCV2 and Chemicals

The APS (>80%) used in the mice was provided by Nanjing Jingzhu Biotechnology Co. Ltd. (Nanjing, China). The APS (>95%) used in cell experiments was purchased from PharmaGenesis, Inc. (America). Tunicamycin (TM) was purchased from Sigma (America). T-AOC, GSH, SOD, and MDA assay kits were obtained from Jiancheng Biotechnology (Nanjing, China). The total protein assay kit was purchased from the Biyuntian Company (Nanjing, China).

### Animals and feeding experiment

The study was carried out according to protocols approved by the Animal Care and Use Committee of Nanjing Agricultural University (Certification No.: SYXK (Su) 2011–0036).

For the first randomized mouse trial, mice were randomly divided into five groups of 10 mice: a normal control group, a PCV2 model group, and three APS groups with doses at 100, 200 and 400 mg APS/kg body weight. The PCV2 infection model was induced by intraperitoneal injection of 1000 TCID50 of PCV2 14 d after APS treatment. The normal control group received equal amounts of 0.5% CMC-Na. Every day during the experimental period, the mice in the APS groups were administered APS in 0.5% CMC-Na by gavage, and normal control mice and PCV2 model mice received equal amounts of 0.5% CMC-Na.

For the second randomized mouse trial, mice were randomly divided into five groups of 10 mice: a normal control group, a PCV2 model group, a PCV2 + 200 mg/kg APS group, a PCV2 + TM group and a PCV2 + TM + 200 mg/kg APS group. The treatment of the control and APS groups was the same as that described above. TM was injected 3 days before PCV2 inoculation (TM were diluted into 0.3 μg/μL, only 3 μg per animal testing injection quantity, namely injected mice with approximately 0.1 mg TM/kg, at 11 d after APS treatment). Each mouse in the PCV2 infection groups received an intraperitoneal injection of 1000 TCID50 of PCV2 14 d after APS treatment. At the end of 4 weeks, the mice were killed; the lobes from each lung were fixed in 5% formalin for 2–3 days, and the tissues were dehydrated through a graded alcohol series, embedded in paraffin, and sectioned at a thickness of 4 μm.

### Measurement of GSH, T-AOC, SOD, and MDA

Blood was rapidly collected from the mice, placed at 37 °C for half an hour, and then centrifuged at 3000× *g* and 4 °C for 10 min. Lung and liver samples were weighed and homogenized with nine volumes of ice-cold normal saline (NS). The homogenates were centrifuged at 5000× *g* for 5 min. The extracted supernatant was saved and stored at 4 °C. The activities of T-AOC and SOD and the levels of GSH and MDA in the serum and tissues were determined according to the instructions of commercial assay kits.

### Histopathological Examination and Immunohistochemical Staining

Liver, spleen and lung samples were collected from the mice and fixed in 10% neutral buffered formalin for haematoxylin-eosin (H&E) staining using standard procedures. For immunohistochemical staining, the tissues were incubated with a monoclonal antibody for the Cap protein and then incubated with streptavidin−peroxidase complex. The peroxidase conjugates were subsequently visualized using diaminobenzidine (DAB) solution.

### Quantitative Real-Time PCR

Quantitative real-time PCR was performed to determine both the number of PCV2 DNA copies and the mRNA levels of ERS related genes in mice (GRP78 and GADD153/CHOP) and PK15 cells (GRP78). For PCV2 measurements, DNA was extracted from PK15 cells or mouse tissues using the TaKaRa DNA Mini kit (TaKaRa, China), and the purified DNA was used as a template for PCR amplification, which was assayed with SYBR Green real-time PCR. A 117 bp fragment from the ORF2 gene of PCV2 was amplified with specific primers (the forward and reverse primers were 5′-TAGTATTCAAAGGGCACAG-3′ and 5′-AAGGCTACCACAGTCAG-3′). Quantitative real-time PCR was carried out using the ABI Prism Step One Plus detection system (Applied Bio systems, USA). A recombinant pMD19 plasmid vector (TaKaRa) containing a PCV2 genome insert as a reference and a TaKaRa SYBR- Green real-time PCR kit (TaKaRa, China) were used.

The relative mRNA levels of GRP78 and GADD153/CHOP in mouse lung tissues and GRP78 in PK15 cells were quantitatively determined using real-time PCR. Total RNA was isolated from the tissues frozen at −70 °C using the RNAiso Plus reagent (TaKaRa) according to the manufacturer’s instructions. The primer sequences for the analysis of GRP78, GADD153/CHOP and GAPDH (a control housekeeping gene) in mice and GRP78 and β-actin (a control housekeeping gene) in pigs are shown in Table 1 and were synthesized by Invitrogen (Shanghai, China). PCR was carried out using the ABI Prism Step One Plus detection system (Applied Bio systems, USA). Potential DNA contamination in the extraction was eliminated using the DNA-Free kit (TaKaRa), and the RNA quality was assessed by the 260/280 nm absorbance ratio. First-strand cDNA was synthesized (TaKaRa, China) according to the manufacturer’s instructions. PCR was carried out using the ABI Prism Step One Plus detection system (Applied Biosystems, USA). Reactions were performed as described in the kit instructions. Each reaction was performed as three replicates. Relative mRNA levels were calculated using the 2^−ΔΔCt^ method^45^ and were normalized to GAPDH or β-actin.

### Western Blot

Tissues (0.1 g) were added to 1 mL lysis buffer and then homogenized; PK15 cells in 6-well cell culture plates were harvested into 100 μl/well of lysis buffer containing 1 mM protease inhibitors (Beyotime, China) and disrupted with sonication. After the above treatments, the lysate was centrifuged at 12,000 rpm for 20 min at 4 °C, and the supernatant fluid was collected. The protein concentration was determined using a BCA kit (Beyotime, China). Fifty micrograms of protein were diluted in sample loading buffer and heated at 95 °C for 5 min. The denatured proteins were resolved by 10% sodium dodecyl sulphate-polyacrylamide gel electrophoresis (SDS–PAGE) and transferred to polyvinylidene difluoride (PVDF) membranes using a semidry transfer cell (Bio-Rad Trans-Blot SD). The membranes were incubated for 40 min at RT in Tris-buffered saline (TBS) containing 5% BSA and 0.1% Tween 20 (TBST) to prevent nonspecific binding and then incubated overnight with specific primary antibodies: anti-Cap, anti-GRP78, anti-CHOP, or anti-β-actin (the CHOP and GRP78 antibodies were obtained from Cell Signaling Technology). After three washes in TBST, the membranes were incubated with an HRP-conjugated secondary antibody (polyclonal anti-rabbit/mouse–horseradish peroxidase from Sigma) diluted in blocking buffer for 40 min at RT followed by three washes. The blots were visualized using a standard enhanced chemiluminescence system (Bio-Rad).

### Cell culture, PCV2 infection, TM or TUDCA treatment

PK15 cells without PCV infection were provided by the China Institute of Veterinary Drug Control and were cultivated in Dulbecco’s minimal Eagle’s medium (DMEM, Invitrogen, USA) supplemented with 4% foetal bovine serum (FBS), streptomycin (20 mg/mL), and penicillin (20 mg/mL) in a humidified atmosphere. PCV2 was generated and stored as described in a previous method^28^.

PK15 cells were pretreated with 15 μg/mL APS or 0.2 mg/mL TUDCA for 10 h. Then, the cells treated with APS were treated with 1.0 μg/mL TM, the medium was removed, and fresh basal medium containing fresh TM and/or 15 μg/mL APS was added to the cells. The PK15 cells were infected with PCV2 and incubated with TM and/or APS for 48 h prior to determination.

### Indirect Immunofluorescence Assay (IFA)

PK15 cells were washed with PBS containing 0.1% Tween 20 (PBST) and fixed in 4% paraformaldehyde. After three washes, the cells were perforated with 0.1% Triton X-100 and then blocked in PBST containing 1% bovine serum albumin (BSA) at 37 °C for 45 min to prevent nonspecific binding. Subsequently, the cells were incubated at 37 °C for 1 h with pig anti-PCV2 antibody (Univ Biotech, China) diluted in PBST containing 1% BSA (PBSTB) (1:50), and after three washes with PBST, a FITC-conjugated rabbit anti-pig antibody (Sigma; diluted 1: 100 in PBSTB) was added and incubated for 1 h at 37 °C. After three washes, the cells were examined under a fluorescence microscope. Cells positive for PCV2 viral antigens were counted in six fields of view.

### Statistics

The data were analysed statistically using the SPSS computer program for Windows (version 19.0). The statistical analyses performed included one-way ANOVA followed by *Duncan*’s multiple range tests. The results were expressed as the mean ± standard error (SE). P-values < 0.05 were considered statistically significant.

## Additional Information

**How to cite this article**: Xue, H. *et al*. Astragalus polysaccharides attenuate PCV2 infection by inhibiting endoplasmic reticulum stress *in vivo* and *in vitro. Sci. Rep.* **7**, 40440; doi: 10.1038/srep40440 (2017).

**Publisher's note:** Springer Nature remains neutral with regard to jurisdictional claims in published maps and institutional affiliations.

## Supplementary Material

Supporting Information

## Figures and Tables

**Table 1:** Primers used for real-time PCR.

Target gene	Gen Bank Accession no.	Primer sequence (5′–3′)	Product, bp
GAPDH (mice)	AC_000042.1	Forward: ATGTTCCAGTATGACTCCAACGCTC	153
		Reverse: GAAGACACCAGTAGACTCCACGACA	
GRP78 (mice)	NC_000068.7	Forward: CACGTCCAACCCGAACGA	182
		Reverse: ATTCCAAGTGCGTCCGATG	
GADD153/C HOP (mice)	NM_001142643.2	Forward: CATGAACAGTGGGCACCATC	163
		Reverse: GCTGGGTACACTTCCGGAGAG	
β-actin (Porcine)	DQ845171.1	Forward: CTGCGGCATCCACGAAACT	147
		Reverse: AGGGCCGTGATCTCCTTCTG	
GRP78 (Porcine)	X92446	Forward: AATGGCCGTGTGGAGATCA	114
		Reverse: GAGCTGGTTCTTGGCTGCAT	
