# Supplementary material for: Astragalus polysaccharides attenuate PCV2 infection by inhibiting endoplasmic reticulum stress in vivo and in vitro
Source: Sci Rep. 2017 Jan 10;7:40440. doi: 10.1038/srep40440 (PMC5223157; doi:10.1038/srep40440)
Supplement: Supporting Information [file srep40440-s1.doc]

**Astragalus polysaccharides attenuate PCV2 infection by inhibiting endoplasmic reticulum stress *in vivo* and *in vitro***

Hongxia Xue^#^, Fang Gan^#^, Gang Qian, Junfa Hu, Shu Hao, Jing Xu, Xingxiang Chen, Kehe Huang*

*College of Veterinary Medicine, Nanjing Agricultural University, Nanjing 210095,*

*Jiangsu Province, China*

Email addresses:

1. Hongxia Xue^#^: 1055491221@qq.com

2. Fang Gan^#^: ganfang@njau.edu.cn

3. Gang Qian: 2014207025@njau.edu.cn

4. Junfa Hu:2013107085@njau.edu.cn

5. Shu Hao: 827594281@qq.com

6. Jing Xu: 2014107089@njau.edu.cn

7. Xingxiang Chen: cxx@njau.edu.cn

8. Kehe Huang*: [khhuang@njau.edu.cn](mailto:khhuang@njau.edu.cn)

^#^ These authors contributed equally to this work and should be considered co-first authors

^*^Correspondence to: Dr. Kehe Huang, College of Veterinary Medicine, Nanjing Agricultural University, Nanjing 210095, Jiangsu Province, China

Tel: +86-25-84395507

Fax: +86-25-84398669

E-mail address: [khhuang@njau.edu.cn](mailto:khhuang@njau.edu.cn)

**Supporting Information**

**Figure S1**


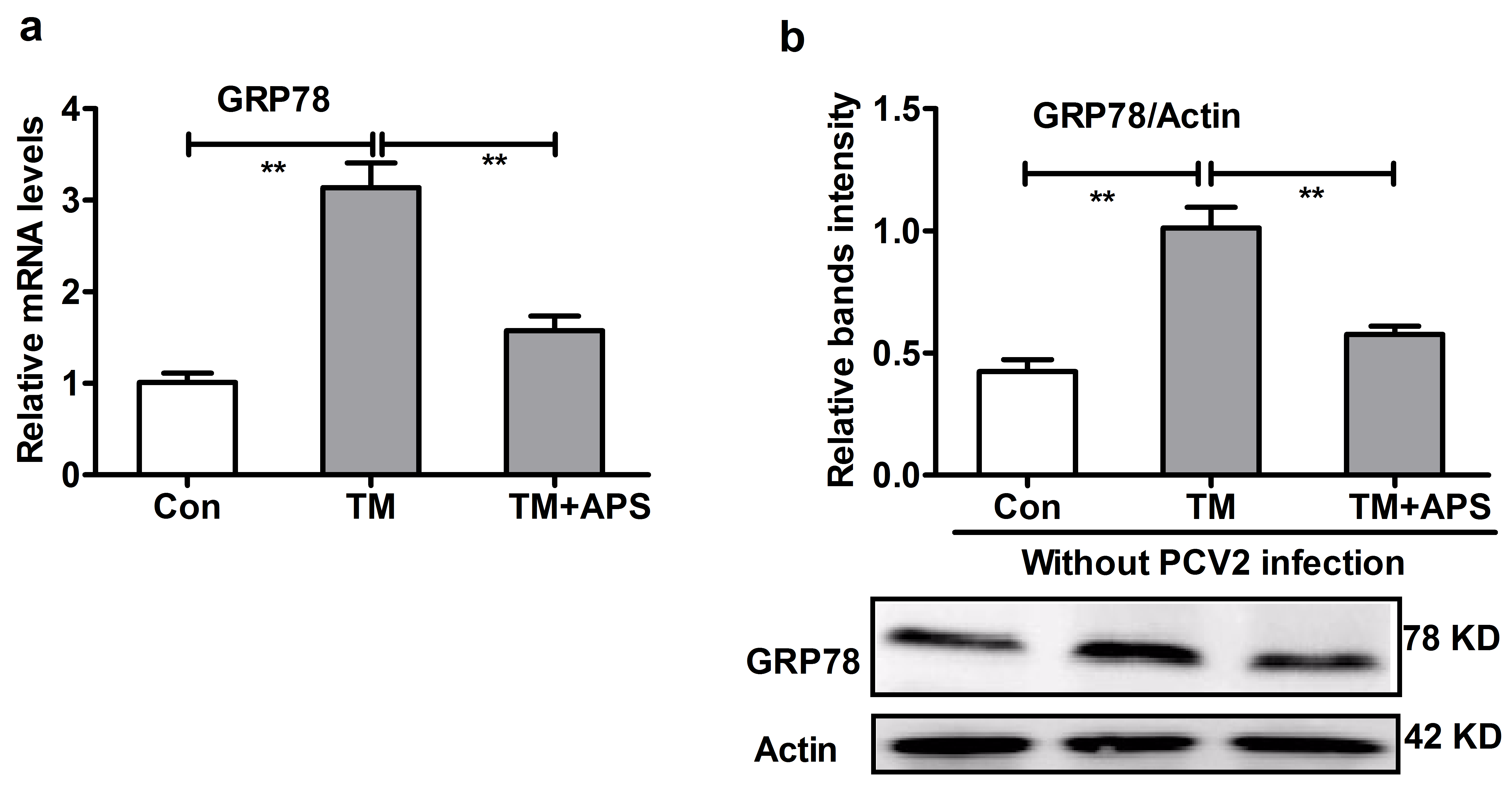


Figure S1 APS decreased GRP78 expression induced by TM in PK15 cells without PCV2 infection. The GRP78 mRNA levels (a) and the relative protein expression (western blot) (b) were assayed as described in the Materials and Methods section. Western blots were analysed under the same experimental conditions. The values shown are the means ± SD from three independent experiments. Groups were compared with a one-way ANOVA followed by a least-significant difference test. *p < 0.05 indicates significance and **p < 0.01 indicates extreme significance.
